# Phylogenetic relationships of the supercontig of sodium channel subunit I (NaV) in 17 species of *Anopheles* (Diptera: Culicidae)

**DOI:** 10.1590/0037-8682-0701-2021

**Published:** 2022-10-24

**Authors:** Valéria Silva Santos, Leticia Cegatti Bridi, Míriam Silva Rafael

**Affiliations:** 1 Instituto Nacional de Pesquisas da Amazônia, Pós-Graduação em Genética, Conservação e Biologia Evolutiva, Manaus, AM, Brasil.; 2 Instituto Nacional de Pesquisas da Amazônia, Laboratório de Vetores da Malária e Dengue, Manaus, AM, Brasil.

**Keywords:** Anopheles, Phylogenetic analysis, Bayesian inference

## Abstract

**Background:**

Malaria is a global health problem and is transmitted by the *Anopheles* species. Due to the epidemiological importance of the genus, studies on biological, phylogenetic, and evolutionary aspects have contributed to the understanding of adaptation, vector capacity, and resistance to insecticides. The latter may result from different causes such as mutations in the gene that encodes the sodium channel (*NaV*).

**Methods:**

In this study, the NaV subunit I scaffold of 17 anopheline species was used to infer phylogenetic relationships of the genus *Anopheles* using Bayesian inference. The evolutionary phylogenetic tree of the *NaV* gene was aligned in the AliView program and analyzed utilizing Bayesian inference, using the software MrBayes.

**Results:**

The anophelines were grouped into five well-supported clusters: 1 - *Anopheles darlingi* and *Anopheles albimanus*; 2 - *Anopheles sinensis* and *Anopheles atroparvus*; 3 - *Anopheles dirus*; 4 - *Anopheles minimus, Anopheles culicifacies, Anopheles funestus, Anopheles maculatus*, and *Anopheles stephensi*; and 5 - *Anopheles christyi, Anopheles epiroticus, Anopheles merus, Anopheles melas, Anopheles gambiae, Anopheles coluzzii*, and *Anopheles arabiensis.*

**Conclusions:**

The topology confirms the phylogenetic relationships proposed in studies based on the genome of some anophelines and reflects the current taxonomy of the genus, which suggests that *NaV* undergoes selection pressure during the evolution of the species. These data are useful tools for inferring their ability to resist insecticides and also help in better understanding the evolutionary processes of the genus *Anopheles.*

## INTRODUCTION

Malaria is a parasitosis caused in humans by five species of parasites (*Plasmodium vivax*, *Plasmodium malariae, Plasmodium ovale, Plasmodium falciparum*, *Plasmodium knowlesi*, and *Plasmodium simium*)^1^ ^,^ ^2^. According to the World Health Organization^3^, malaria is a public health problem, with 229 million recorded cases of the disease around the world. Of these, 90% occurred in Sub-Saharan Africa, with an estimated 409,000 deaths, which include children younger than five years in 99 countries in Africa, Asia, and Latin America. 

This disease is transmitted by mosquitoes of the genus *Anopheles*, which belongs to the Culicidae family. The subfamilies Anophelinae and Culicinae present evolutionary divergence over 100 million years, which occurred simultaneously, and are thus sister groups phylogenetically speaking^4^ ^,^ ^5^. Mosquitoes of the genera *Aedes* and *Culex* are grouped in the subfamily Culicinae, while those of the genus *Anopheles* belong to the subfamily Anophelinae. The genera *Bironella* and *Chagasia* also belong to this subfamily^6^ ^-^ ^8^.

In Africa, the main vectors of human malaria parasites are *Anopheles gambiae*, *Anopheles arabiensis*, and *Anopheles funestus*; the corresponding main vectors are *Anopheles albimanus* and *Anopheles pseudopunctipennis* ^9^ in Mexico and Central America and *Anopheles stephensi* and *Anopheles culicifacies* in Asia. In South America, the vectors involved with malaria transmission belong to the subgenus *Nyssorhynchus* (*Anopheles darlingi, Anopheles aquasalis, Anopheles nuneztovari, Anopheles oswaldoi, Anopheles triannulatus, Anopheles tadei, Anopheles konderie* complex, and *Anopheles albitarsis)* ^9^ ^-^ ^11^ and *Kerstezia* (*Anopheles cruzii* and *Anopheles bellator*)^12^. *Anopheles darlingi* is the main vector of disease-causing parasites in South America, with geographical distribution covering the eastern Andes, Colombia, Venezuela, Bolivia, Peru, Paraguay, Argentina, Guianas, and Brazil; however, it is absent in the extreme northeastern parts of Brazil^9^ ^,^ ^13^.

Population studies of *An. darlingi* in relation to its role as the main transmitter of human malaria in Brazil have demonstrated that geographical differences apparently do not interfere with its vector capacity^14^ ^-^ ^16^, even if one considers that *An. darlingi* may comprise a complex of 3 well-structured species^17^. Strategies for combating malaria have two main focuses: prevention, through the control of mosquito vectors, and case management^3^ ^,^ ^14^ ^,^ ^18^. The development of insecticides with long residual effects was one of the most important advances for their application in public health. Dichloro-diphenyl-trichloroethane (DDT) was the first insecticide with a prolonged residual effect and is an organochloride that was developed during the Second World War^19^, but which has had its use suspended due to its persistence in the environment. Pyrethroids have been used in the control of malaria vectors due to their rapid action against the insect’s nervous system and low toxicity in mammals^20^. These insecticides are mainly used in indoor residual spraying and to control agricultural pests^14^ ^,^ ^18^.

Pyrethroids and DDT and its analogs are neurotoxic, act in axonic transmission, and share a similar mechanism of action as voltage-dependent sodium channel (NaV) modulators^21^. They interact with the sodium channels distributed along the axon, prolonging or preventing their normal closure after the transmission of the nerve impulse and allowing an excessive flow of Na^+^ ions into the interior of the nerve cell, leading to paralysis of the central and peripheral nervous systems^21^.

Mosquito resistance to pyrethroid insecticides potentially represents the greatest threat to the implementation of malaria prevention programs^22^. This category of resistance is called knockdown resistance (kdr) and results from specific mutations in the gene that encodes the sodium channel as it changes its affinity to insecticides, and is observed in several insects such as *Musca domestica* ^23^.

The decreased sensitivity of the target site of action of pyrethroids and also DDT in *An. gambiae* has been described in association with two alternative substitutions to a single codon of the sodium channel gene. The first results in the replacement of a residue (L1014F) of leucine (TTA), which is present in the wild allele, by phenylalanine (TTT) in the amino acid position of the gene encoding the trans-membrane subunit (S6 of domain II) of the sodium channel. This mutation in *An. gambiae* is widely dispersed in West Africa^22^ ^,^ ^24^. The second involves a replacement (L1014S) of the leucine residue (TTA) by serine (TCA) in the same amino acid position, and is found in East Africa^25^ ^,^ ^26^.

In addition to *An. gambiae*, two more mutations (L1014C and L1014W) have been reported in two Asian populations of *Anopheles sinensis*, which change the amino acid leucine to cysteine and tryptophan, respectively. In addition, at the site immediately preceding the classical kdr mutation, N1013S substitution occurs, which changes the amino acid asparagine to serine^27^. In populations of *An. culicifacies* from India, in addition to the L1014F/L1014S substitutions, a new mutation was described at site 1010, involving a replacement of valine with leucine (V1010l)^26^. A comparison of the *NaV* gene sequence in different insect species showed that this sequence is highly conserved. However, different numbers of exons are observed among different species^28^.

To date, NaV mutations have been described in at least 13 different species of anophelines. *An. gambiae*, which is the most studied mosquito, presents three mutational variants (L1014F, L1014S, and N1575Y) in 20 of the 54 African countries, in addition to *An. arabiensis*, which presents two variants (L1014F and L1014S), present in seven countries of the African continent^28^.

Due to its importance as a vector, the genus *Anopheles* has been subjected to many studies to determine its biological characteristics; however, its molecular and evolutionary characteristics need to be studied further. Sequencing the genome of some species, such as *An. darlingi*, fills some gaps in the knowledge about their genes and has opened up several possibilities, including a contemplation of the evolutionary history of anophelines. It also provides valuable information that can lead to new strategies for reducing malaria transmission^29^.

The literature contains few studies on the molecular phylogeny of *Anopheles* mosquitoes, though one can cite that of Neafsey *et al*.^4^, which generated a phylogenetic tree of 16 *Anopheles* mosquitoes and other Diptera, as outgroups, using the maximum-likelihood method. In addition to this study, Harbach & Kitching^8^, using cladistics, reviewed the phylogenetic relationships among anopheline species.

Although there are studies of phylogenetic trees of *Anopheles*, there are no records about the evolutionary behavior of a single gene or contigs with gaps (scaffolds) in these mosquitoes. Therefore, we conducted a phylogenetic study using the scaffold sequence (subunit I gene) of *NaV* to contribute to a greater understanding of the biological and molecular characteristics such as adaptation, resistance to insecticides, and vector capacity.

## METHODS

The scaffold sequence (subunit I) of the sodium channel of *An. darlingi*, fosmid clone, inserted in the pCMV SPORT6 cloning vector (Invitrogen, Waltham, MA, USA) was obtained from the genome of this mosquito^29^. The sequence has been deposited in the Vector Base under the accession number ADAC000755 (5,066 bp). From this sequence, 37 orthologs for the supercontig (scaffold) gene *NaV* were recorded. These 37 sequences were analyzed according to the following criteria: in the first, sequences that did not belong to the mosquitoes of the family Culicidae were excluded; in the second, the number of copies of the gene, the query, and the target of 16 anophelines were recorded, excluding sequences that had values lower than 60% in relation to *An. darlingi*. For this analysis, two *Aedes* species were used as the outgroup (Table 1). Then, the program AliView^30^ was used, which uses the software MUSCLE (Multiple Sequence Comparison by Log-Expectation)^31^ to align the sequences.

**TABLE 1:** Orthologous sequences to the supercontig sodium channel for *Anopheles darlingi* (ADAC000755) compared to the 16 species of *Anopheles*, *Aedes aegypti*, and Aedes *albopictus* (outgroup), according to Gene Ontology (GO).

Mosquito species	Accession number	Target ID%	Query ID%	Ontology		
				Biological process	Molecular function	Cellular component
*Anopheles albinamus*	AALB008211	91.42%	97.53%			
*Anopheles arabiensis*	AARA004284	84.70%	99.04%			
*Anopheles atroparvus*	AATE017690	88.73%	95.84%			
*Anopheles christyi*	ACHR003796	87.40%	98.19%			
*Anopheles coluzzii*	ACOM036769	88.51%	79.22%			
*Anopheles culicifacies*	ACUA006175	87.72%	96.39%			
*Anopheles dirus*	ADIR010287	89.60%	99.10%	GO:0006811	GO:0005216	GO:0005891
*Anopheles epiroticus*	AEPI000310	86.83%	96.51%	GO:0055085	GO:0005245	GO:0016020
*Anopheles funestus*	AFUN020364	88.68%	98.61%	GO:0070588	GO:0005509	
*Anopheles gambiae*	AGAP002577	88.82%	99.04%			
*Anopheles maculatus*	AMAM018441	99.55 %	26.93%			
*Anopheles melas*	AMEC011109	97.97%	92.83%			
*Anopheles merus*	AMEM015017	97.50%	63.43%			
*Anopheles minimus*	AMIN004000	87.51%	89.94%			
*Anopheles sinensis*	ASIS008573	95.75%	97.77%			
*Anopheles stephensi*	ASTE000510	90.31%	94.88%			
*Aedes aegypti*	AAEL027127	85.30%	95.06%			
*Aedes albopictus*	AALF005277	92.29%	63.43%			
*Anopheles darlingi*	ADAC000755	-	-			

**Source:** https://vectorbase.org/vectorbase/app, accessed: 09/11/2021.

When inputting data into the BEAST software^32^, the nucleotide substitution model was accessed, providing a better fit to a set of aligned sequences. In this analysis, the software JModelTest^33^ and PAUP^34^ were used jointly. The pattern identified for NaV sequences was the General Time Reversible, which considers the different substitution frequencies from one base to the other. The run was carried out with 10,000 repetitions. The phylogenetic tree of the *NaV* gene was generated and visualized in the FigTree program^35^ for the 17 species of anophelines and two species of *Aedes* (outgroup).

## RESULTS

**FIGURE 1:**
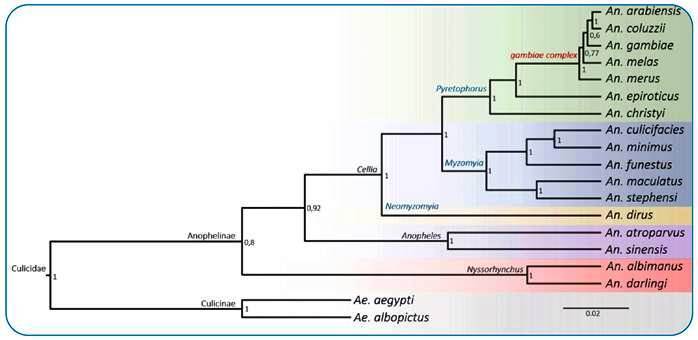
Topology of the *NaV* gene phylogenetic tree, with 17 species of anophelines and two species of *Aedes* (outgroup).

The 17 anophelines were grouped into five well-supported clusters (Figure 1). The species of the subgenus *Cellia*, which formed the most diverse clade, were grouped into three clusters: 1 - *Anopheles dirus* (*Neomyzomyia* series); 2 - *Anopheles minimus, Anopheles culicifacies, An. funestus, Anopheles maculatus*, and *An. stephensi* (*Myzomyia* series); and 3 - *Anopheles christyi, Anopheles epiroticus, Anopheles merus, Anopheles melas, An. gambiae, Anopheles coluzzii*, and *An. arabiensis* (*Pyretophorus* series)*.* In the latter, the species of the *An*. *gambiae* complex is present, representing the most efficient vectors of malaria in Africa. In the other cluster, which is monophyletic and contains the subgenus *Anopheles*, *An. sinensis* and *Anopheles atroparvus* were grouped. Finally, in the most basal and also monophyletic cluster (subgenus *Nyssorhynchus*), *An. darlingi* and *An. albimanus* were grouped.

The bootstrap values in each branch indicate that the NaV phylogeny was generated with high reliability since values below 0.7 suggest that the sequences have a high degree of similarity. In general, the topology results obtained confirm the same phylogenetic relationships that were proposed in studies based on genomes of some anophelines^4^ ^,^ ^8^ and also reflect the current taxonomy of the genus *Anopheles*, which suggests that *NaV* undergoes selection pressure during species evolution.

## DISCUSSION

From the supercontig sequences of the subunit I of the sodium channel gene, the generated phylogenetic and evolutionary trees of the *NaV* gene showed a topology with some coincidences in relation to the phylogenetic trees for some species of anophelines^4^ ^,^ ^5^ ^,^ ^8^. In the topologies presented by these authors, as well as in the evolutionary trees of the *NaV* gene, *An. darlingi* and *An. albimanus* appeared as sister groups. Coincidence was also recorded in the grouping of sequences of the *NaV* gene of some species of the *An. gambiae* complex (*An. merus, An. melas, An. coluzzii*, and *An. gambiae*). The sequence of the *NaV* gene of *An. christyi* was grouped with the sequences belonging to the complex *An. gambiae* (*An. merus, An. melas, An. gambiae, An. coluzzii*, and *An. arabiensis*); this grouping also occurred in the topologies of the phylogenetic trees^4^ ^,^ ^5^ ^,^ ^8^. 

The NaV tree generated in this study also provides a good representation of the trees of species found in the literature for the *Anopheles* species in Asia and Oceania^5^.

The NaV sequences of *An. sinensis* and *An. atroparvus* are closely related in evolutionary terms to the NaV sequence of *An. dirus*, *An. stephensi, An. funestus, An. culicifacies, An. maculatus*, and *An. minimus*. The latter are evolutionarily grouped into a clade since they are sister groups^4^ ^,^ ^8^. 

The values in each branch refer to the statistical bootstrap resampling test^36^, which infers the reliability of the branches based on generations. The NaV phylogenetic tree was generated with high reliability, and it was acknowledged that bootstrap values below 70% are too low to consider a true branch and, above 90%, the branch is considered to have a high degree of support. The overall reliability of the tree is 99% according to the data generated in the BEAST software program information file.

In the clade where *An. melas, An. gambiae, An. coluzzii*, and *An. arabiensis* (*Pyretophorus* series) are found, there are bootstrap values close to and below 70%. It is suggested that this occurs due to the high degree of similarity between the sequences. To reduce doubts about the topology of the NaV tree, these data were also subjected to maximum-likelihood analysis. The topology found was the same (data not shown), which corroborates the findings of this work for the *NaV* gene tree. Differences were found in the positions of *An. christyi* and *An. epiroticus* in comparison to the result obtained by Neafsey et al.^4^, who found *An. christyi* to be more related to the species in the *An. gambiae* complex. Instead, in the tree of the *NaV* gene, we found that *An. epiroticus* was related to the species that occur in Africa (*An. gambiae* complex).

It is important to bear in mind that the evolution of the sodium channel gene can occur in a different process of species evolution. This may justify the differences found between the clades of the evolutionary phylogenetic tree of the *NaV* gene in the 17 species of anophelines in this study. Although the tree of a single gene, such as the *NaV* gene, cannot represent the evolutionary history of species of a genus, the genome evolves conservatively, and this demonstrates that species may be closely related, when the gene trees and their coincidences in the evolutionary history of considered species are analyzed^37^ ^,^ ^38^. It is suggested that the sodium channel gene has undergone selection pressure during the evolution of the species, since these mosquitoes may present susceptibility or resistance to neurotoxic insecticides. 

Biological factors, such as habitat and ecological niche, can influence the differentiation of a gene throughout its evolutionary history, justifying possible disparities when studied in isolation^38^. Studies of gene trees within *Anopheles* have contributed to the understanding of parts of these processes that act independently in each gene, and some works present similar topologies when compared to species tree topologies^39^. Most trees generated from genes also have disparities^40^ in the study of the GNBP domain^38^ species of the complex *An. gambiae* which are not grouped in the same clade; however, *An. arabiensis* and *An. quadriannulatus* appear as sister groups, which corroborates this study. Further research is needed to understand characteristics such as adaptation to environmental pressures that have led to the evolutionary success of genes and the *Anopheles* species^38^.

In general, the topology results generated in this study confirm the phylogenetic relationships proposed in studies based on the genome of some anophelines, reflects the current taxonomy of the genus, and indicates that the *NaV* gene undergoes selection pressure during the evolution of the species. These findings may help infer the ability to develop resistance to insecticides and, also, gain a better understanding of the evolutionary processes within *Anopheles*.
